# A case report of spontaneous coronary artery dissection complicated by stent fracture

**DOI:** 10.1097/MD.0000000000016612

**Published:** 2019-08-30

**Authors:** Hanxiang Gao, Ye Hu, Qi Li, Suyu Yao, Zheng Zhang, Ming Bai

**Affiliations:** Department of Cardiology, First Hospital of Lanzhou University, Lanzhou, Gansu, China.

**Keywords:** intravascular ultrasound, percutaneous coronary intervention, spontaneous coronary artery dissection, stent fracture

## Abstract

**Rationale:**

Spontaneous coronary artery dissection (SCAD) is a rare, complex disease, and nowadays poorly understood. The overall incidence of SCAD ranges from 0.28% to 1.1% in angiographic studies. Therefore, the true incidence of SCAD is most likely underestimated due to asymptomatic or sudden cardiac death before diagnosis. Stent fracture (SF) is a multifactorial issue. Longer vessel remodeled by 2 stents can be more prone to have SF due to higher radial force.

**Patient concerns:**

In this paper we report a 48-year-old man with chest pain for 2 years.

**Diagnoses:**

Elective coronary angiography revealed a linear dissection in obtuse marginal branch (OM).

**Interventions:**

He underwent percutaneous coronary intervention (PCI) with the guidance of intravascular ultrasound (IVUS).

**Outcomes:**

Then SF was revealed 9 months later.

**Lessons:**

This is the first case report of SF after coronary intervention therapy in SCAD patients.

## 1 Introduction

Spontaneous coronary artery dissection (SCAD) is a rare, complex disease, and nowadays poorly understood. The overall incidence of SCAD ranges from 0.28% to 1.1% in angiographic studies.^[1]^ Therefore, the true incidence of SCAD is most likely underestimated due to asymptomatic or sudden cardiac death before diagnosis.^[2]^ Stent fracture (SF) is a multifactorial issue. Longer vessel remodeled by 2 stents can be more prone to have SF due to higher radial force.^[3]^ In this paper we report a 48-year-old man with a linear dissection in obtuse marginal branch (OM). He underwent percutaneous coronary intervention (PCI) with the guidance of intravascular ultrasound (IVUS), and then SF was revealed 9 months later. This is the first case report of SF after coronary intervention therapy in SCAD patients.

## 2 Case report

**Figure 1:**
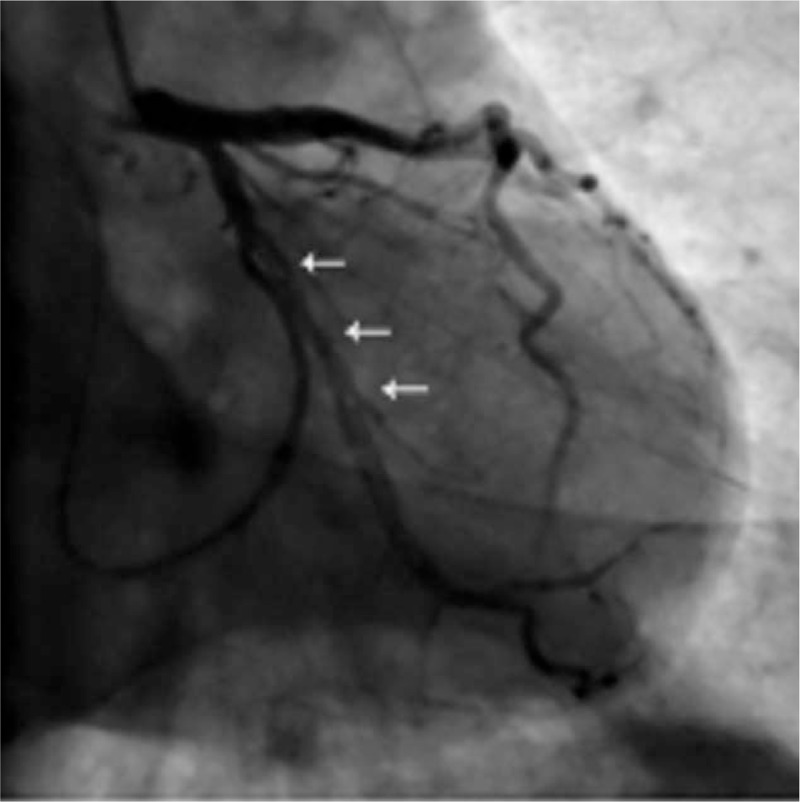
Coronary angiography showed a linear dissection in the OM extending from the proximal to mid-distal end (arrow) with moderate stenosis at the middle. OM = obtuse marginal branch.

**Figure 2:**
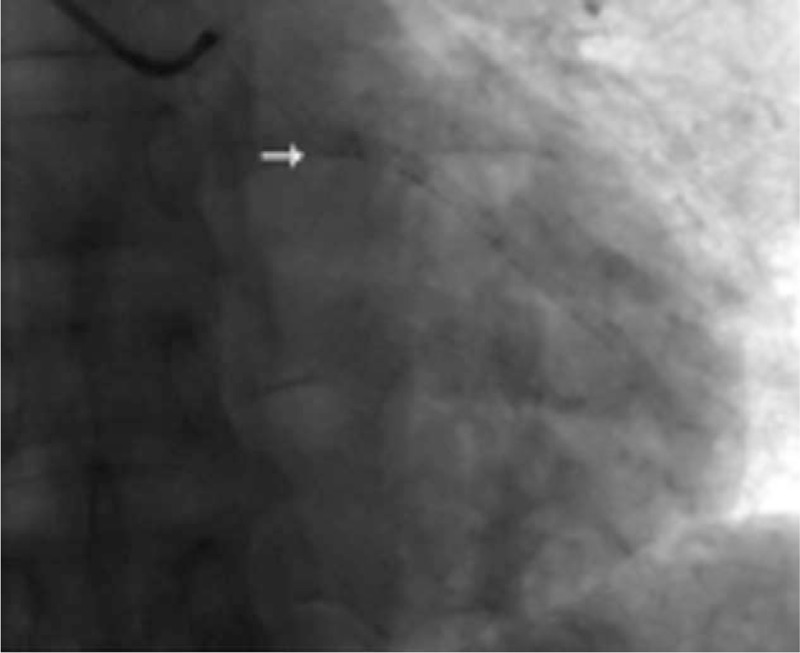
Follow-up coronary angiogram showing stent fracture of OM with acquired transection with gap in the stent body. OM = obtuse marginal branch.

Here we present a case of a 48-year-old male who was referred to our institute due to intermittent chest pain for 2 years which worsened in the last week. His only cardiovascular risk factor is tobacco use for 30 years. No significant electrocardiogram and troponin I change was detected. Elective coronary angiography revealed a linear dissection in OM (Fig. 1) and the others were completely normal. In the next 3 days the patient suffered from chest conditions twice a day. Then PCI was performed. A 6F EBU 3.5 guiding catheter (Medtronic Corp, Minneapolis, Minnesota) engaged left main coronary artery successfully. IVUS was carefully performed and revealed the lesion clearly. After the guidewire was passed to the distal OM, IVUS was used again to make sure the guidewire was in the true lumen. Subsequently, OM was predilated and 2 drug-eluting stents (DES) (2.5 × 36 mm and 2.75 × 24 mm EXCEL, JW Medical Systems, Yantai, Shandong, CHN) were implanted in overlapping, being followed by postdilatation. Then IVUS revealed a satisfactory angiographic result. He was instructed to take daily aspirin 100 mg, clopidogrel 75 mg, atorvastatin 20 mg, and captopril 25 mg after discharge. He got stabbing chest pain again 9 months later. The repeat angiography revealed the fracture of the proximal stent with coronary flow maintained well (Fig. 2). What is more, the right coronary artery was 70% diseased and proximal anterior descending branch had a tight stenosis. Then the patient was evaluated with a treadmill stress test and the results were negative. He was thus discharged receiving aspirin, atorvastatin, and isosorbide mononitrate. At 3-year follow-up, he still experienced atypical chest discomfort almost every day, which was described as mild and transient and resolved spontaneously. Further work-up was denied due to the patient requests.

## 3 Discussion

In fact, the precise etiology and the mechanism of SCAD are uncertain. Prospective series revealed that SCAD associated with coronary artery disease, which is more common in men.^[4]^ However, classic cardiovascular risk factors are unlikely to be related to SCAD except cigarette smoke.^[5]^ Coronary atherosclerotic plaques were detected in the patient for the first time when he had been treated with sufficient medical therapy for 9 months. Interestingly, the plague mainly occurs in the right coronary artery rather than the stented segment. So the procedural factors could be ruled out. The underline mechanisms are required to be confirmed further.

Optimal treatment strategies for SCAD represent a burning controversial question. Due to its rarity, information on this disorder comes from case reports and retrospective reviews mainly. A retrospective meta-analysis containing 440 cases identified that SCAD patients had a statistically significant better outcome when treated with an early aggressive strategy as compared to a conservative strategy (*P* = .006).^[6]^ Therefore, another 2-year follow-up of 45 SCAD patients managed with conservative therapeutic strategy demonstrated that no patient experienced a myocardial infarction or died suddenly. And complete disappearance of the dissection image occurs in 54% patients.^[4]^ Using of intravascular imaging such as IVUS or optical coherence tomography could facilitate PCI techniques.^[7]^

In this case, we performed PCI under IVUS without any complications. He was doing well until 9 months later a complete SF was revealed. SF is a multifactorial issue. Stent overlap and stent type are possible causes in our patient. Longer vessel remodeled by 2 stents can be more prone to have SF due to higher radial force.^[3]^ And the overlapping portion would be localized rigidity which will create hinge point deforming the stent along with vessel movement.^[8]^ Funnily enough, the fracture in this case locates on exactly the same vessel as the dissection. We also suppose that mild flexion existing in this vessel which is hard to be identified in a 2-dimensional angiography. The torsion injured the arterial wall contributing to the dissected media. Ultimately, this mechanical stress broke down the integrity of stents. On top of the mechanical intervention, appropriate medical options were recommended throughout the treatment. Nevertheless, the cause of rapid plaque progression in left anterior descending artery and right coronary artery remains unclear.

Comparing with bare metal stents (BMS), DES means apparent lower restenosis rates. Therefore, SF occurs less in patients implanted BMS. Perhaps the better neointimal coverage without the protection of drugs enhances the strut. It is also possible that the hyperplasia masked the fractured part entirely.^[9]^ In treating of our patient, the characteristic property of sirolimus-eluted stents (SES) is another possible mechanism. Actually, most cases of SF are accompanied by the use of SES. It is interpreted by several theories. Stent conformability is believed to play a pivotal role in the development of SF. The rigid close-cell structure of SES leads to decreased conformability followed by higher SF rates. And the structure of SES is thicker than other DESs. So, an existed SF in SES could be determined easily.^[10]^ Therefore, the higher incidence did not necessarily mean worse clinical prognosis. Conversely, the rate of in-stent restenosis is lower than paclitaxel-eluting stents.^[9,10]^

This is the first case report of SF after PCI in SCAD patients. Optimal treatment strategy of SCAD remains unclear. Coronary intervention can be considered as a reasonable option. IVUS can ensure the success of this procedure. Conversely, we propose conservative management strategy after the SF was detected. A careful long-term clinical surveillance is required to make sure no major adverse coronary events would occur.

## Author contributions

**Data curation:** Ye Hu, Qi Li.

**Resources:** Suyu Yao.

**Writing – original draft:** Hanxiang Gao.

**Writing – review & editing:** Zheng Zhang, Ming Bai.
